# Rare association of two cardiovascular malformations successfully corrected in a single surgery: a case report

**DOI:** 10.1186/s13019-017-0619-z

**Published:** 2017-07-24

**Authors:** Fu-Yang Mei, Zhi-Xuan Bai, Zhi-Bin Hu, Bing Zhou, Yong Cui

**Affiliations:** Department of cardiothoracic surgery, Zhejiang Provincial People’s Hospital, Hangzhou, 310014 China

**Keywords:** Partial anomalous pulmonary venous connection, Coarctation of the aortic surgery

## Abstract

**Background:**

Partial anomalous pulmonary venous connection (PAPVC) without an atrial septal defect (ASD) associated with coarctation of the aortic arch is a rare congenital cardiac anomaly. This rare combination is only described in a few studies; none report the correction of these two malformations in a single surgery.

**Case presentation:**

A 5-year-old girl was admitted to our hospital because the echocardiography revealed coarctation of the aortic arch; this diagnosis was confirmed by computed tomography (CT), which also showed her left superior pulmonary vein draining into the vertical vein without ASD (Fig. 1). She exhibited no special clinical symptoms. Her upper-limb blood pressure was approximately 110/90 mmHg, whereas her lower-limb blood pressure was approximately 75/50 mmHg.

**Conclusions:**

We surgically repaired the case of PAPVC to the vertical vein with aortic coarctation, in which the two cardiovascular malformations were corrected in a single surgery without cardiopulmonary bypass.

## Background

PAPVC is a condition in which one or more pulmonary veins drain into the right atrium or a systemic vein; PAPVC is a frequently reported congenital cardiac anomaly [1]. However, PAPVC without ASD and with coarctation of the aortic arch is considered a rare congenital cardiac anomaly. To date, no reports describe the correction of both cardiovascular malformations in a single surgery.

## Case presentation

**Fig. 1:**
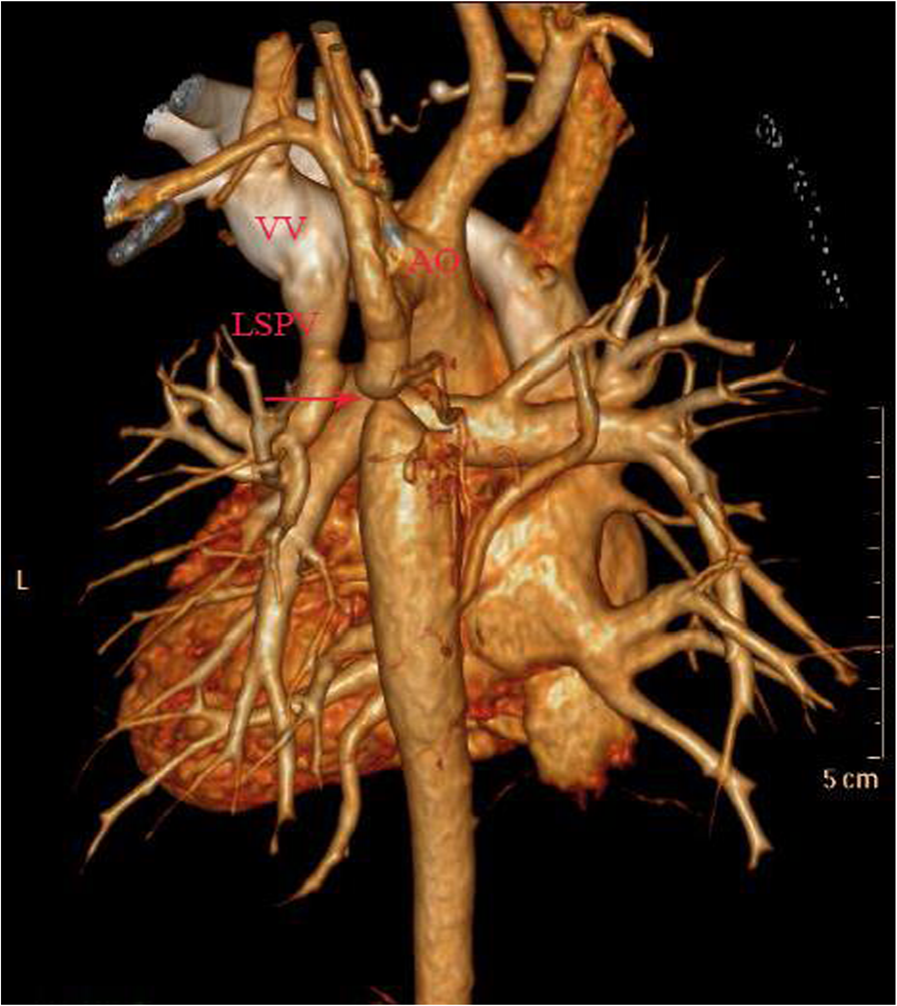
Preoperative computed tomography demonstrating the right superior pulmonary vein draining into the vertical vein and the coarctation of the aortic arch (AO = aorta; VV = vertical vein; LSPV = left superior pulmonary vein; *red arrow* = coarctation of the arch)

A 5-year-old Chinese girl was admitted to our hospital because of her upper-limb high blood pressure. Regular echocardiography revealed coarctation of the aortic arch; this was confirmed by computed tomography (CT), which also showed her left superior pulmonary vein draining into the vertical vein without ASD (Fig. 1). She exhibited no special clinical symptoms and the pregnancy ultrasound screening in the prenatal diagnosis of fetal heart defects of the patient was negative. Her upper-limb blood pressure was approximately 110/90 mmHg, whereas her lower-limb blood pressure was approximately 75/50 mmHg.

**Fig. 2:**
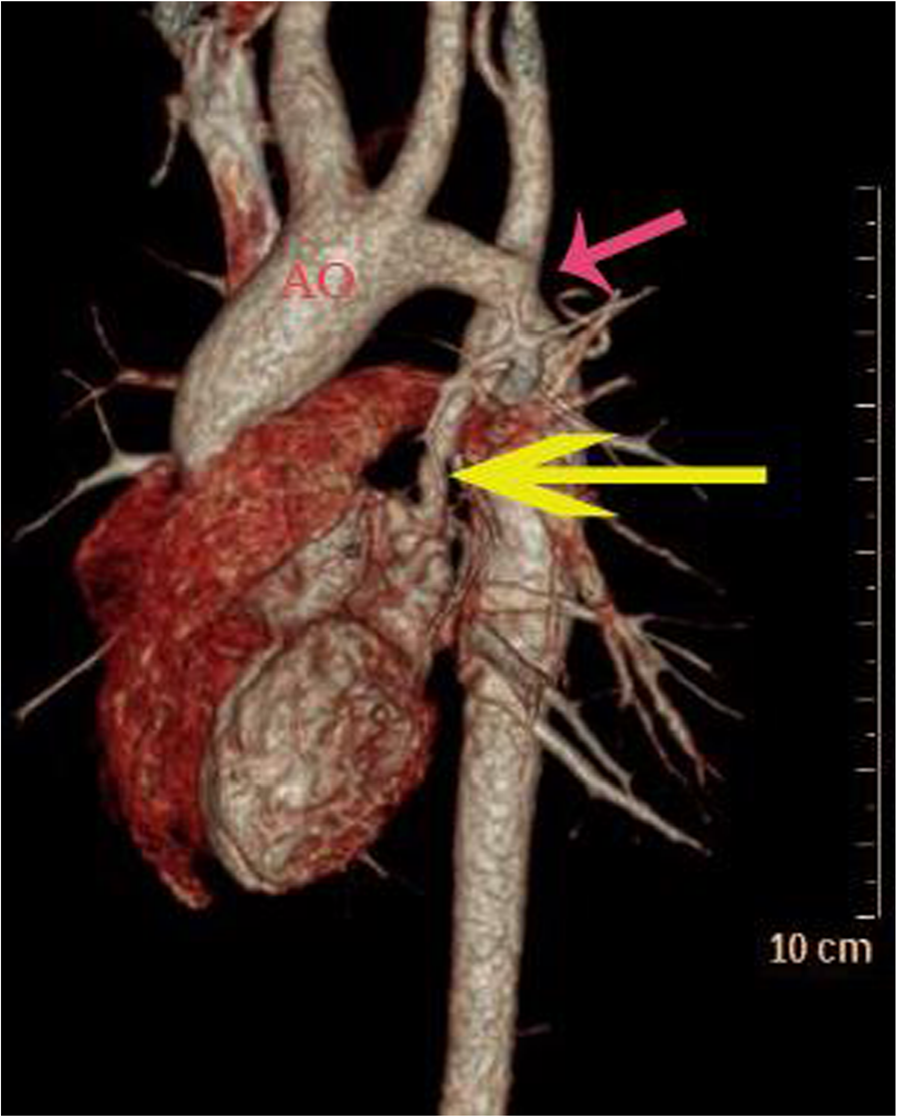
Postoperative computed tomography demonstrating the left superior pulmonary vein draining into the left atrial appendage (AO = aorta; *red arrow* = correction of the coarctation; *yellow arrow* = left superior pulmonary vein draining into the left atrial appendage)

**Fig. 3:**
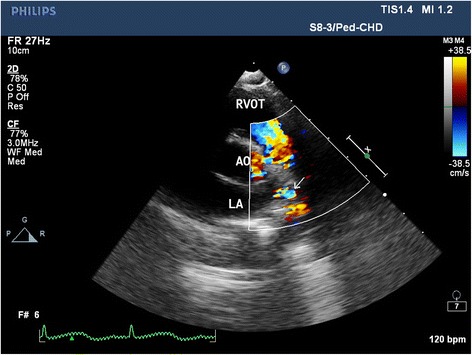
Postoperative echocardiograph demonstrating the blood flow of the aorta and left superior pulmonary vein (LA = left atrium; AO = aorta; RVOT = right ventricular outflow tract; *white arrow* = the left superior pulmonary vein draining into the left atrial appendage)

General anesthesia was induced. Before surgery, we lowered the patient’s body temperature to 35 °C. She was placed in a right lateral position, and thoracotomy was performed through the fourth intercostal space. The aortic arch, anomalous pulmonary vein, descending aorta, and vertical vein were dissected and encircled. After clamping both sides of the coarctation and clipping it out, we sutured the descending aorta with an end-to-end anastomosis. After the coarctation was corrected, we detached the anomalous left superior pulmonary vein from the vertical vein, which we then closed by continuous suturing. Next, we sutured the left superior pulmonary vein to the left atrial appendage. Intraoperative transesophageal echocardiography showed no pressure gradient or laminar flow through the anastomosis after blood flow was resumed. After surgery, her upper-limb blood pressure was approximately 90/60 mmHg, whereas her lower-limb blood pressure was approximately 80/50 mmHg. The patient was successfully extubated in the pediatric intensive care unit approximately 2 h after surgery. During this surgical procedure, we used just one unit of platelets in case of anastomotic leakage. One year after surgery, echocardiography (Fig. 2) demonstrated complete anastomosis of the aorta and the left atrial appendage and there were no pressure gradients both at the site of the coarctation repair and the pulmonary vein re-implantation at the LA. CT scan (Fig. 3) revealed that the forms of the left superior pulmonary vein and descending aorta were improved.

## Discussion

PAPVC is a rare congenital anomaly first described by Winslow in 1789, Pathological studies suggest that this anomaly occurs in 0.7% of the population [2], and has been observed in 0.4%–0.7% of autopsies [3]. Most cases of PAPVC are associated with a sinus venosus defect, and PAPVC from the right lung is twice as common as PAPVC from the left lung [4]. Anomalous pulmonary veins originating from the left lung are reported in only 10%–18% cases [5]; a left superior pulmonary vein draining into the vertical vein without an ASD occurs even less frequently.

The development of the pulmonary veins is a complicated process that occurs early in embryonic life, the most accepted process was that blood returning from the lung buds initially drains into the splanchnic plexus, which communicates with paired cardinal veins as well as umbilicovitelline veins, after superior vena cava and inferior vena cava formation, pulmonary vein arises as an outpouching from the dorsal wall of the left atrium. With time, the common pulmonary vein communicates with the portion of the splanchnic plexus that drains blood flow from the lungs. Pulmonary venous connections to the cardinal and umbilicovitelline veins normally involute, and the common pulmonary vein becomes incorporated into the dorsal wall of the left atrium, ultimately typically giving rise to four separate pulmonary veins, Pulmonary venous developmental anomalies happen if any of these processes fails to occur properly [6, 7].

Coarctation of the aortic arch is a common diagnosis among congenital cardiac defects, accounting for 6–8% of live births with congenital heart disease, with an estimated incidence of 1 in 2500 births [8]. Coarctation of the aortic arch occurred mainly due to an abnormal development of the fourth aortic arch in the fourth in the first 12 weeks of fetal life. Which may be asymptomatic for a long time and was discovered only by chance and very few showing significant cardiovascular or respiratory symptoms requiring treatment [9]. Correction of the anomaly should always involve an extended resection, whenever possible, because the elasticity of aortic tissue allows excessive movement of both segments of the thoracic aorta.This case of PAPVC without an ASD and with coarctation of the aorta is the first to be described in the literature, although details of the surgery required to correct each malformation have been reported independently.

## Conclusions

We successfully performed the surgical correction of the rare combination of PAPVC to the vertical vein and aortic coarctation, in which the two cardiovascular malformations were corrected in a single surgery without cardiopulmonary bypass. This case demonstrates that in patients with aortic and other cardiovascular malformations in the left pleural cavity, thoracotomy can be performed first instead of sternotomy.

## Abbreviations

ASD: Atrial septal defect
PAPVC: Partial anomalous pulmonary venous connection
